# Supplementary material for: 3D visualization of macromolecule synthesis
Source: eLife. 2020 Oct 14;9:e60354. doi: 10.7554/eLife.60354 (PMC7669265; doi:10.7554/eLife.60354)
Supplement: Source code 1. — Source code used in the 3D quantification across scales of a regenerating axolotl humerus depicted in Figures 3 and 4. A tutorial describing the process in detail is provided together with the ImageJ and Matlab scripts. This material is also available on Zenodo at https://doi.org/10.5281/zenodo.3891878. The original image used as the starting point is available at Northeastern University’s Digital Repository and also upon request to the authors. [file elife-60354-code1.zip › Source code file 1/Tutorial_MultiscaleAnalysis_v3.pdf]

3D Visualization of Macromolecule Synthesis

Supplementary material: multiscale analysis of a regenerating axolotl humerus

Timothy J. Duerr[1], Ester Comellas[2,3], Eun Kyung Jeon[1], Johanna E. Farkas[1], Marylou Joetzjer[4], Julien Garnier[4], Sandra J. Shefelbine[3,5], James R. Monaghan[1,*]

[1]Department of Biology, Northeastern University, Boston, MA 02115, USA

[2]Department of Mathematics, Laboratori de Càlcul Numeric (LaCàN), Universitat Politècnica de Catalunya (UPC), 08034 Barcelona, Spain

[3]Mechanical and Industrial Engineering, Northeastern University, Boston, MA 02115, USA

[4]University of Technology of Compiègne, Rue Roger Couttolenc, 60200 Compiègne, France

[5]Department of Bioengineering, Northeastern University, Boston, MA, USA

This document describes in detail the process followed in Fiji [1] and MATLAB [2] to obtain the results shown in Figures 3 and 4 of the main text[1]. It is written as a step-by-step tutorial aimed at inexperienced users of Fiji and/or MATLAB. The scripts mentioned here are also provided as supplementary material and contain self-explanatory annotations of their contents[2]. The original .czi image and additional intermediate files are available upon request to S.J. Shefelbine (s.shefelbine@northeastern.edu).

The multiscale analysis is demonstrated here on an axolotl humerus bone rudiment, but the process is easily adaptable to other organs given that a 3D image stack with staining that allows distinguishing organ shape (AHA in our case) and the cell shape (EdU in our case) is provided.

Table of Contents

Figure 3 results **2**

Figure 3. Import the image stack to process. **2**

Figure 3A. Align image stack along the proximodistal axis of the humerus. **4**

Figure 3B. Segmentation of the humerus. **6**

Figure 3C. Apply the humerus mask on the aligned and cropped image stack. **10**

Figure 3D. Reslice the mask and analyze its cross-sections. **11**

Figure 3E. Cell segmentation and object counting. **13**

Figure 3F. Reslice and mask the rotated cropped image, and analyze the pixel maps. **22**

Figure 4 results **24**

References **25**

---

[1] A preprint version of the main text is available on bioRxiv: https://doi.org/10.1101/2020.06.24.169300.

[2] All versions of this tutorial and the associated files are available on Zenodo: https://doi.org/10.5281/zenodo.3891879.

Figure 3 results

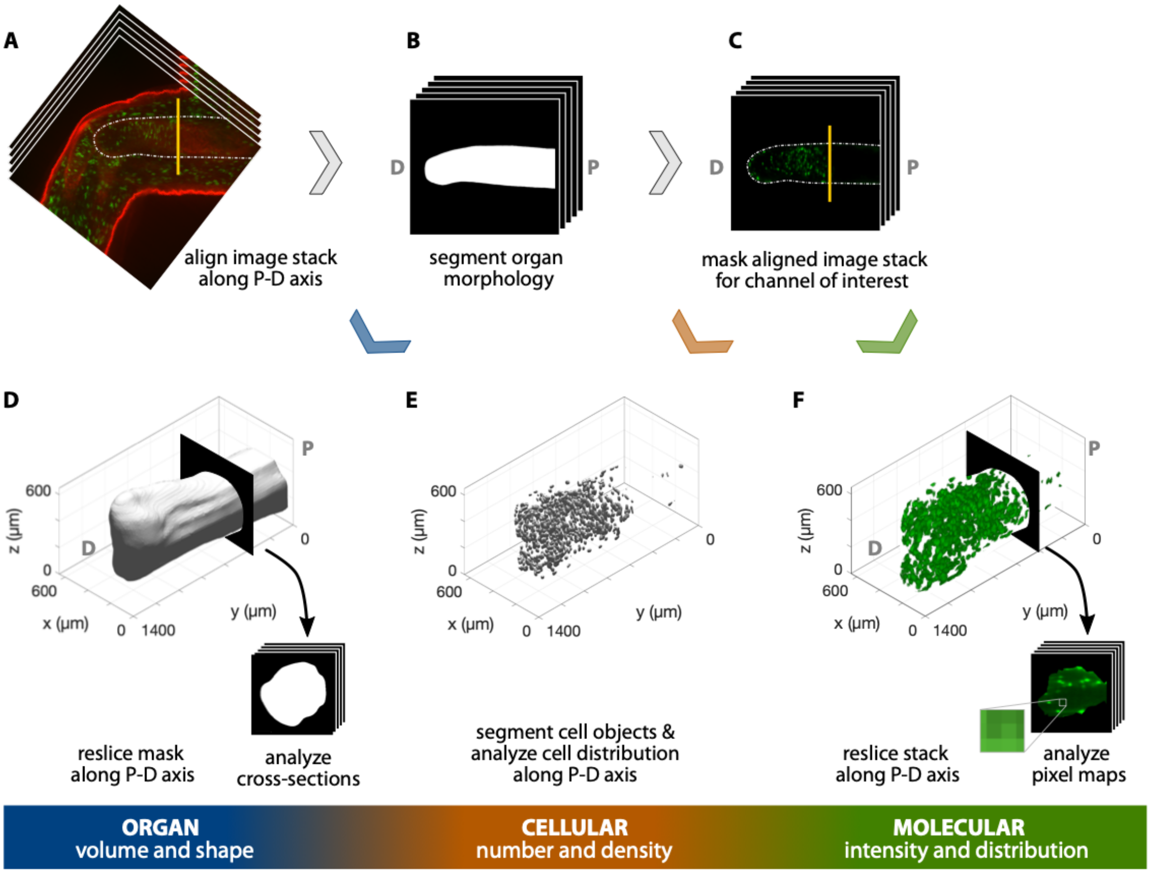

Figure 3. Import the image stack to process.

- Using the Bio-Formats plugin [3] in Fiji, import the .czi file: *Plugins › Bio-Formats › Bio-Formats importer*. We use this plugin instead of dragging and dropping the file on the Fiji toolbar because we must make sure the OME metadata [4] is read correctly.

  Tip: an alternative to using virtual stack when the image is too large for Fiji to load, is to assign more memory, if your computer has the capacity. Type *Memory* in the search window of the tool bar and select *Memory & Threads* and click on *Run*. Change the maximum memory value. As a rule of thumb, select about 75% of your total RAM capacity.

  Also, all commands can be found by typing the name into the search bar, instead of navigating through menus.

  It is useful to keep a window open to keep track of the memory usage while you work in Fiji. Go to (*Plugins › Utilities › Monitor Memory...*) If you double-click on the Memory window, you might see the usage go down. This activates the garbage collector, freeing memory that is not being used.

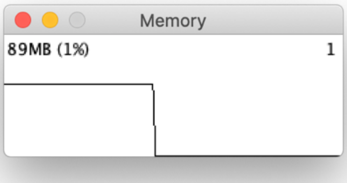

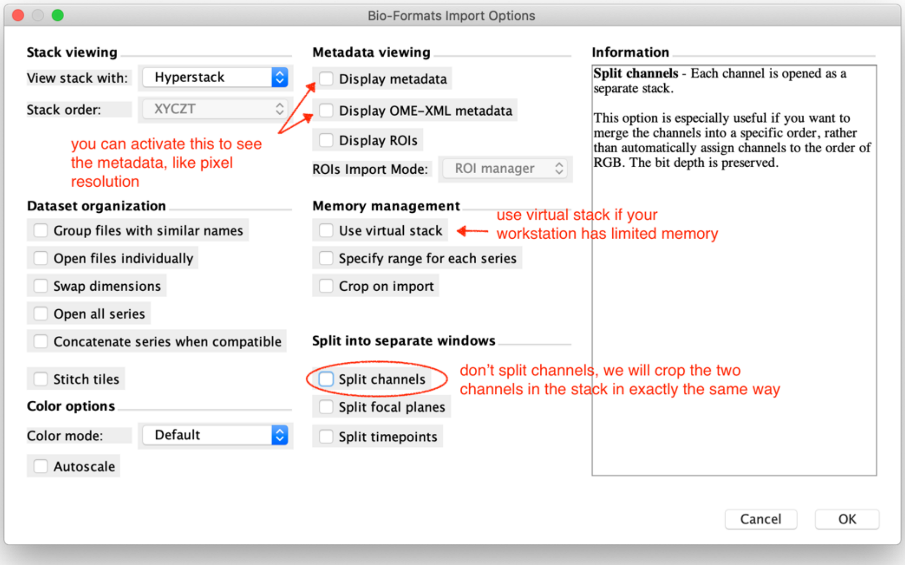

- Take note of the pixel resolution (x, y and z values). We will need this later on in the MATLAB code and, possibly, during the processing in Fiji. Certain operations remove the resolution properties of the stack. If you did not display metadata with the import or you want to check at any time the pixel resolution, you will find it in (*Image › Properties*).

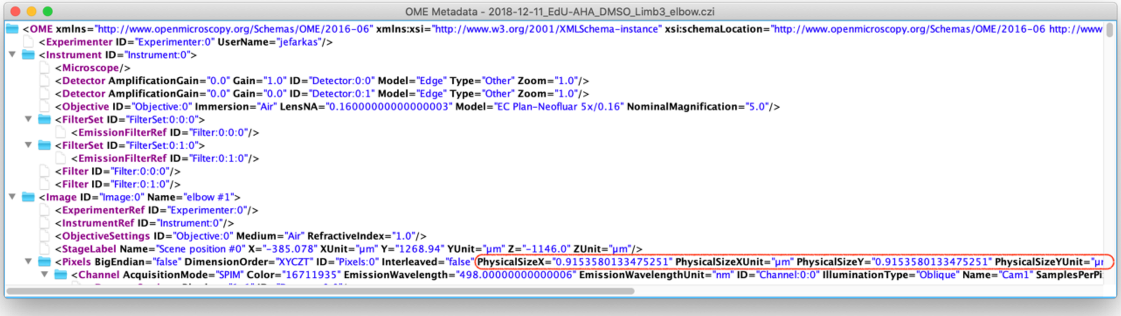

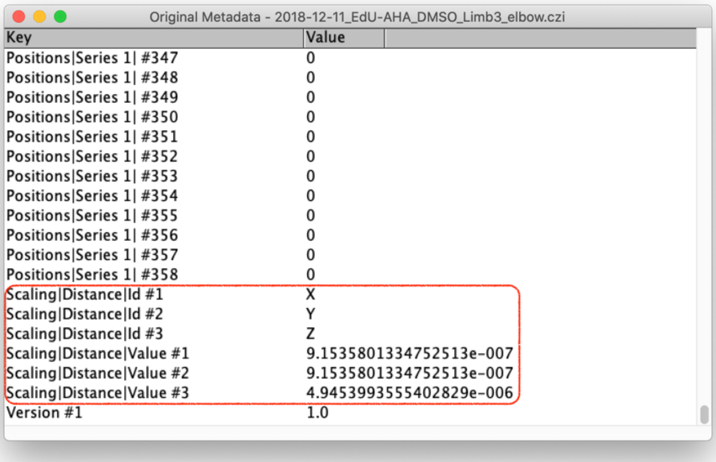

- The image may appear very dark. To adjust the visualization, go to (*Image › Adjust › Brightness/Contrast*) and click on *Auto* in the window that appeared.

  Tip: You might need to scroll to a central slice in the stack for the auto-adjustment to work better. You can also set the limits of the display range manually in *Set*. Do not click on *Apply* or the limits set will be permanently applied on the image stack, modifying the intensity of the data we want to analyze.

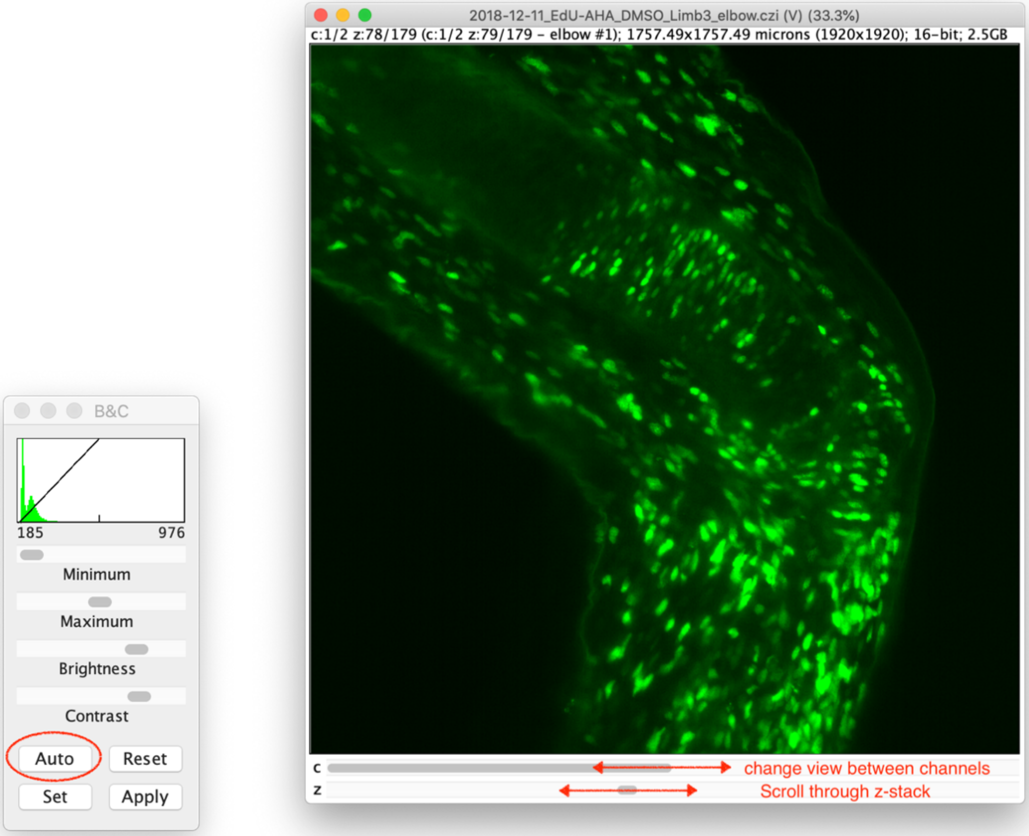

Tip: Save the stack as a .tif file. Most of Fiji's operations do not have an "undo" option, so it is good practice to save files (*File › Save As… › Tiff…*) at each step and work always on duplicate copies (*Image › Duplicate…*). To ensure the OME data is all retained and stored correctly, instead of saving the stack export as an .ome.tif using Bio-Format exporter.

Figure 3A. Align image stack along the proximodistal axis of the humerus.

- Rotate the image stack (*Image › Transform › Rotate…*) to vertically align the humerus' proximodistal axis. Activate *Preview* and adjust the angle until the humerus bone rudiment is vertical. Grid lines will help with the adjustment. We use a bicubic interpolation and choose to enlarge the image to keep as much humerus as possible. We use the AHA channel because the bone rudiment geometry is clearer, and rotate the stack 54 degrees. Click on *Yes* to process the whole stack.

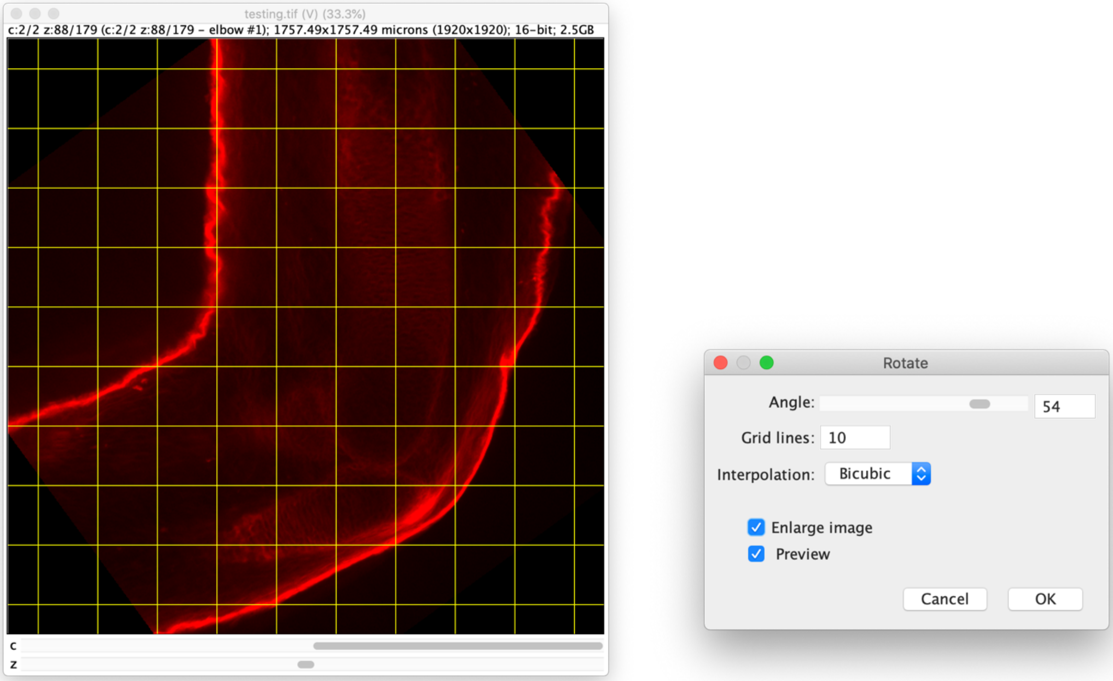

- Crop the image stack to reduce file size. We will only analyze the humerus, so we can create a rectangle around it and duplicate the stack. In our example, we use a rectangle with top left corner at position (1324, 344), width of 544 and height of 1524 (values given in "pixels"). We can also remove the slices in which the rectangle is "empty", at the top and bottom of the stack. So, we keep only slices 10-140 when duplicating.

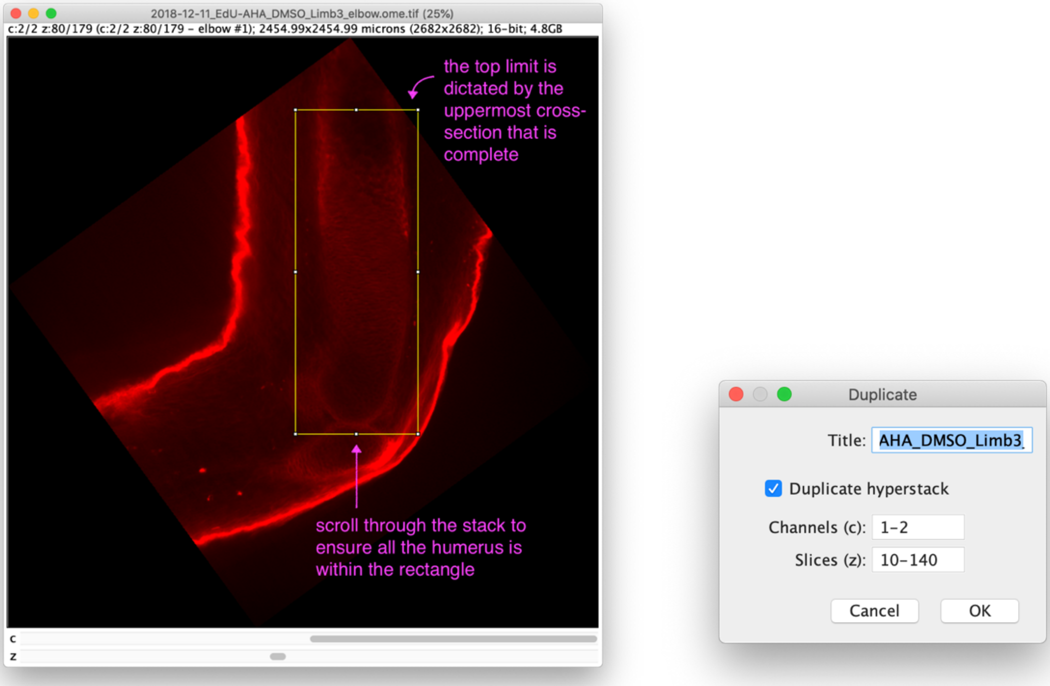

- It is a good idea to save the resulting stack, if you have not done so yet. Now we can split the channels (*Image › Color › Split Channels*; or import the saved stack and click on *split*

channels in the importer window). We did not split channels until now, to ensure we applied the exact same rectangle for cropping both channels.

Figure 3B. Segmentation of the humerus.

Tip: The outcome of the segmentation will depend greatly on the person doing the segmentation. It is important that a same person performs it and is consistent throughout the process, particularly if several samples are to be processed and compared. To ensure results were as consistent as possible, we segmented the same rudiment three separate times and verified that difference between results was within a 10% variation. We used the whole histogram for comparison.

- Open the AHA (red) channel in the Segmentation Editor (*Plugins › Segmentation › Segmentation Editor*) to create the mask. We set the *Brightness & Contrast* to min=750 and max=3500 to help distinguish better the outline of the bone rudiment. Switching the visualization from red to grayscale might also help.

  Here we include a summary and tips on how we segmented the bone rudiment, but do check out the ImageJ [5] documentation for more comprehensive instructions: https://imagej.net/Segmentation_Editor.

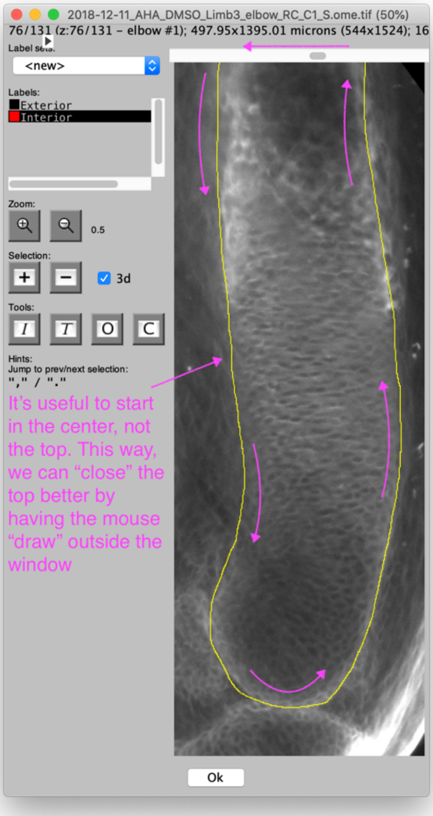

Use the "bean" icon to draw the outline of the humerus. Then, scroll several slices and draw again. Then, click on "I" (interpolate) to generate shapes in the slices in between. The 3D checkbox should be on.

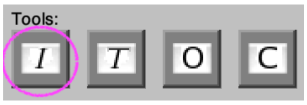

"T" (threshold) refines the selected area based on a locally adjusted threshold. "O" (open) and "C" (close) applies the corresponding kernels to the selection and can be used for smoothing (see video in documentation, minutes 0:46 and 1:25.).

Then, add the shapes as mask. Make sure to check "3d" so all slices are added.

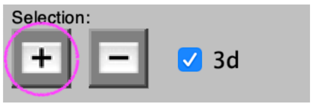

A new window with the file extension renamed ".labels" will appear with the masks in each slice.

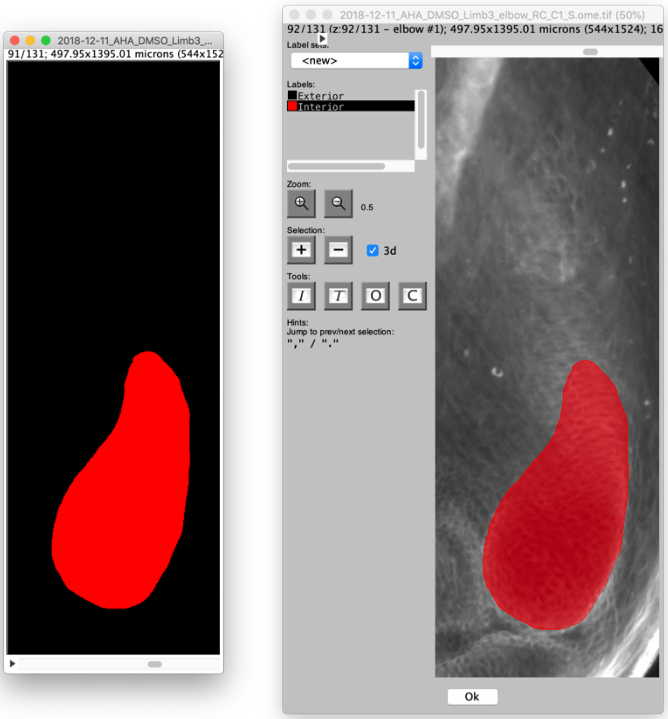

If the interpolation didn't work out well, you can add/remove parts of the mask. Just draw new shapes and click the "+" or "-" button. However, it is better if one anticipates this and manually draws outlines in strategic slices, for example the one with the most downward position of the tip.

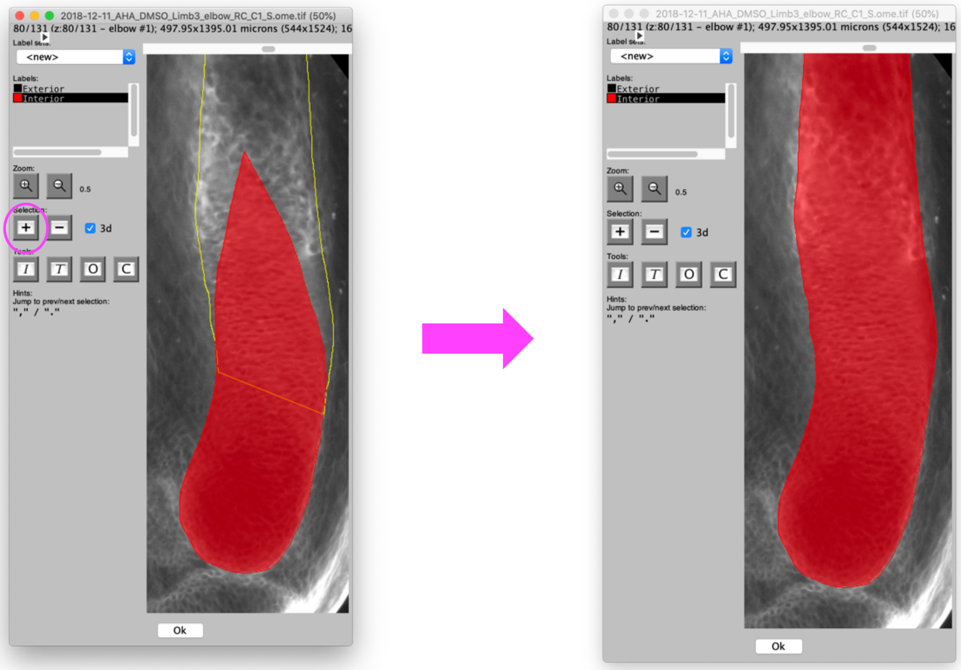

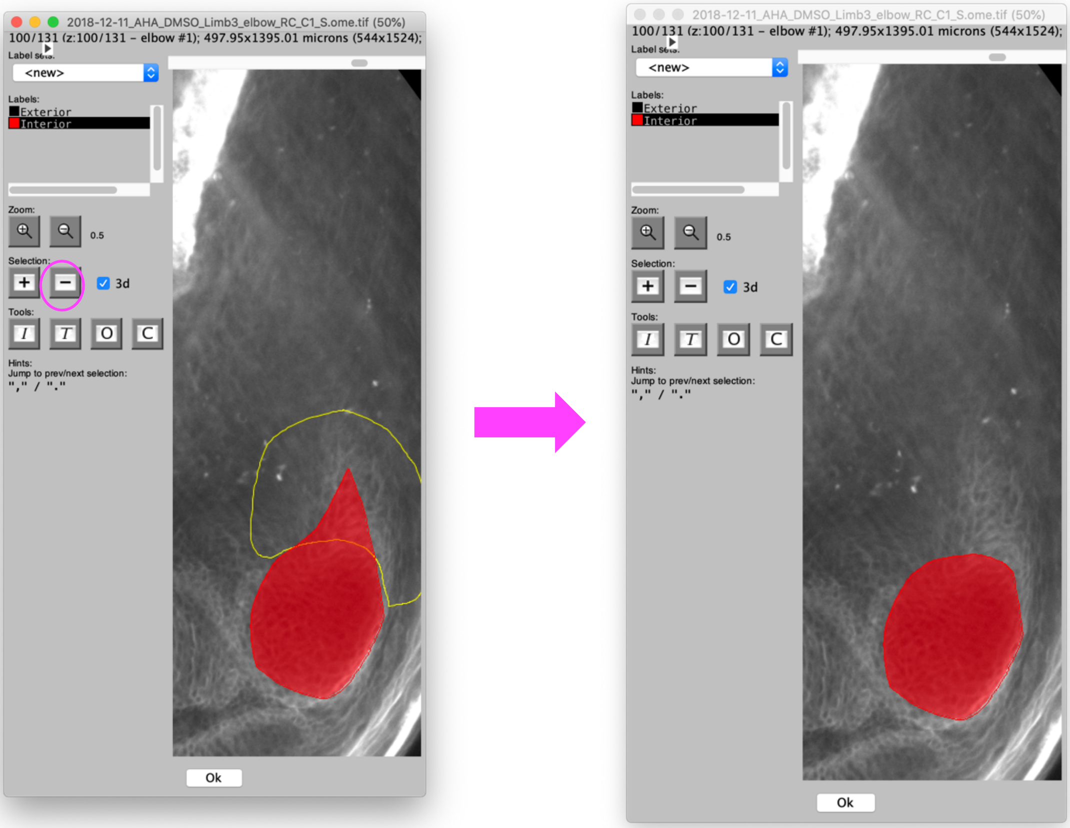

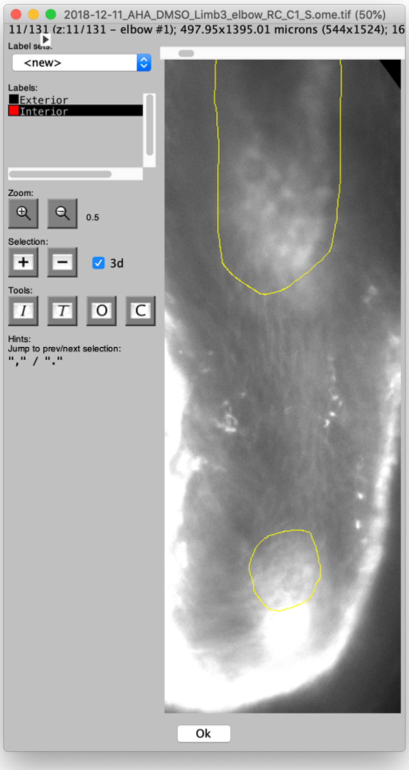

Tip: Press the "shift" key to draw separate shapes in a same slice. The software is able to interpolate between one shape in a slice and two shapes in a slice several slices beyond.

Also, it's better to use the interpolation function smartly than to segment each slice manually. In manual segmentation of consecutive stacks, we can't draw the shapes exactly in the same position as before. So, our 3D surface will not be as smooth and look "uneven":

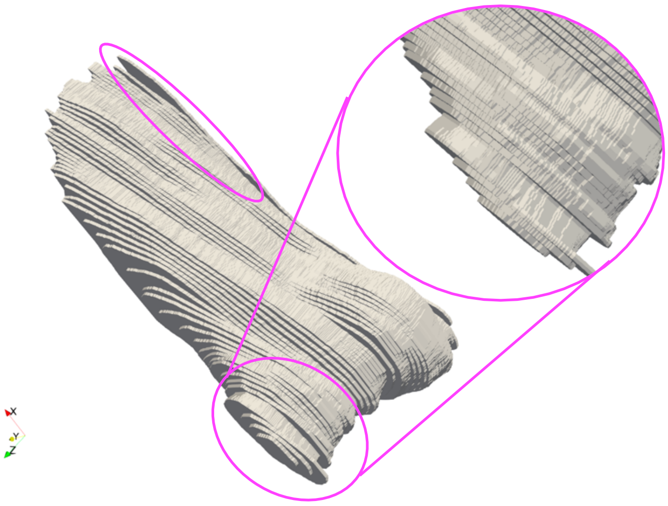

- Once you're happy with the results, save the "labels" stack. This is our mask. To convert it to a 3D surface, open the 3D Viewer plugin [6]. Then export the surface as an .stl. First, convert the volume to a surface (*Edit › Display As… › Surface*) and then export (*File › Export Surfaces … › STL (binary)*). It doesn't really matter if you choose ASCII or binary, they will contain the exact same information. A binary file occupies less space and is preferable. If your 3D volume is "squashed", the pixel resolution was probably lost at some point in this process. Check the properties of the image stack with the mask and update the values (we took note of them at the beginning when we looked at the metadata of the .czi file).

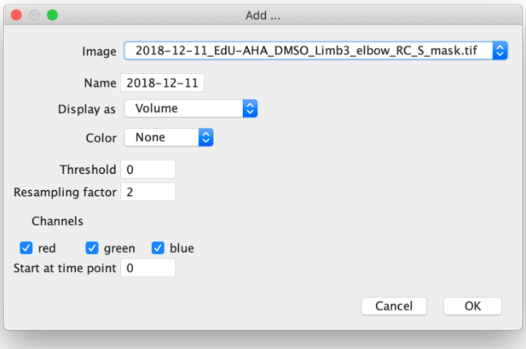

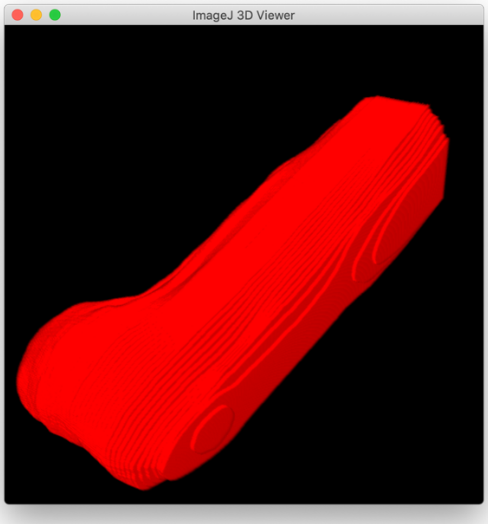

For data analysis purposes, we will use this stack of masks without further modification. However, for visualization purposes, we can smooth the surface so the slices are not visible. For that, we use MeshLab [7]. We open the .stl file clicking on *Unify Duplicated vertices*.

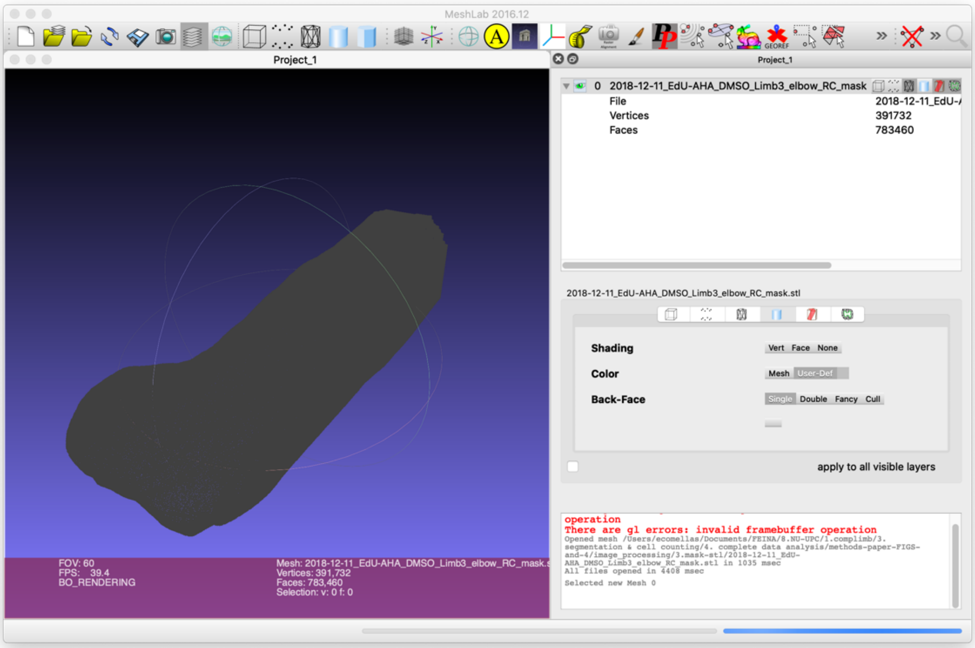

We then reduce the file size and smooth out the surface by simplify the mesh using Clustering Decimation (*Filters › Remeshing, Simplification and Reconstruction › Simplification: Clustering Decimation*) with the following properties:

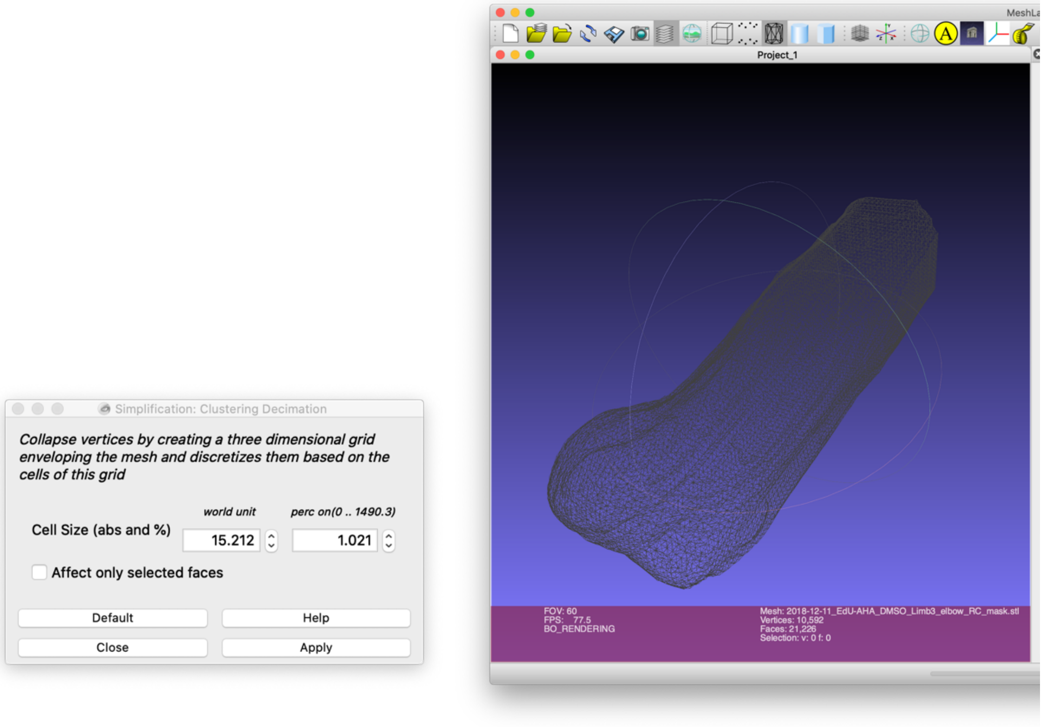

We can further smooth the surface using HC Laplacian smoothing. We can repeatedly apply these two filters in succession, until we get a nice shape for visualization.

This process has modified the volume and cross-sections that we just segmented, this is the reason why we choose not to use the simplified surface for our analysis, to minimize unnecessary manipulation of our data.

Save the Humerus_mask-smooth.stl file (or another filename of your choice) for visualization in MATLAB.

Figure 3C. Apply the humerus mask on the aligned and cropped image stack.

There are several ways of masking the aligned and cropped image stack, which we split into two separate channels. Here we explain one.

- Open the stack containing the mask, and the stack with the image you want to crop. They should both be vertically aligned and cropped in exactly the same manner so that the overlay will coincide.

Note: First, ensure that your 8-bit mask has "0" intensity pixels in the background and "1" intensity pixels in the mask. This is easy to check by hovering the mouse pointer on the image and reading the value in the Fiji tool bar. The image stack we will crop should still be a 16-bit image! If this changed, we must redo the processing and ensure we don't lose this information.

- Use the Image Calculator (*Process › Image Calculator*) to multiply the two image stacks. Repeat the process for the other channel.

  Tip: duplicate and rename each stack before applying the calculator.

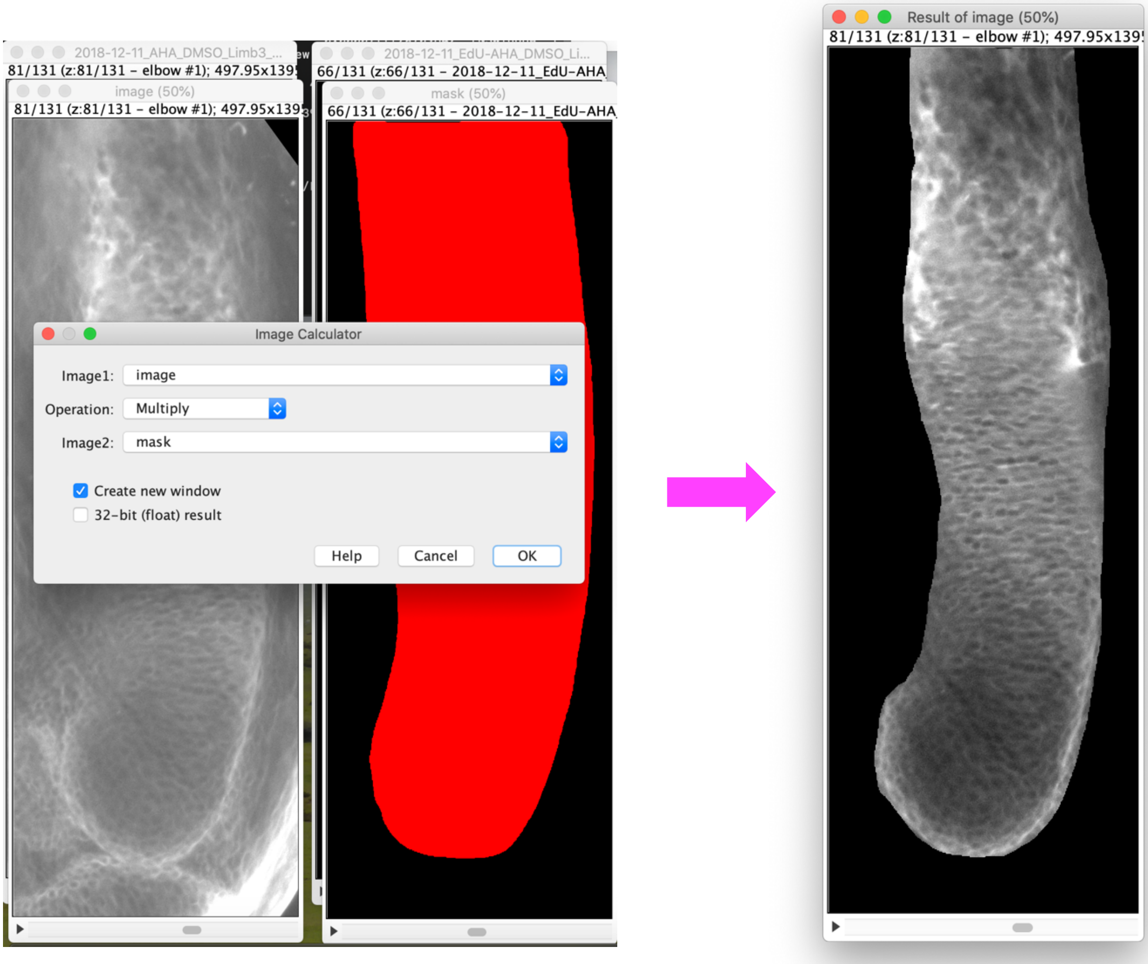

Figure 3D. Reslice the mask and analyze its cross-sections.

- We use the mask created in Figure 3B to analyze the shape and volume of the organ with BoneJ [8]. We want to study cross-sections along the proximodistal axis, so we must reslice the mask perpendicular to that axis (which is the vertical axis in the mask we created). First, double-check once again that the pixel resolutions are correct, go to (*Image › Stacks › Reslice [/]...*) and select *Start at Top* and *Avoid interpolation*. The output spacing should be the z resolution of the pixels.

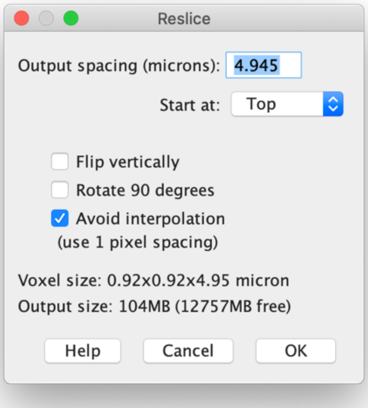

- We can then duplicate the result, creating a substack such that we delete the top incomplete slices and the bottom empty slices (keep only 10–1425, in our case). Save this new stack as a .tif file.

- Now, we obtain the geometry data from the BoneJ plugin (*Plugins › BoneJ › Slice Geometry*). Untick all options except *Process stack*. If you want to see the minimum and maximum axis of inertia of each slice in the 3D Viewer, select *3D Annotation*. Our bone has value 1 (contents of the mask) and our background has value 0, so change the values accordingly. The humerus might look a bit too long, but it is only a visualization effect: check the properties and the spacing between slices is 0.9153586 microns (= voxel depth).

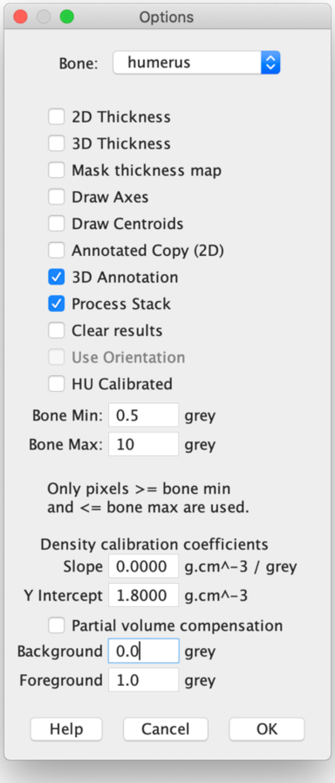

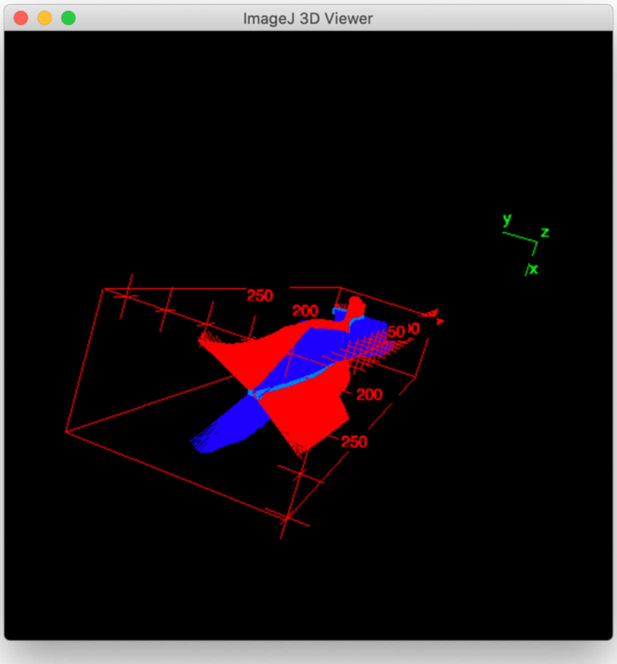

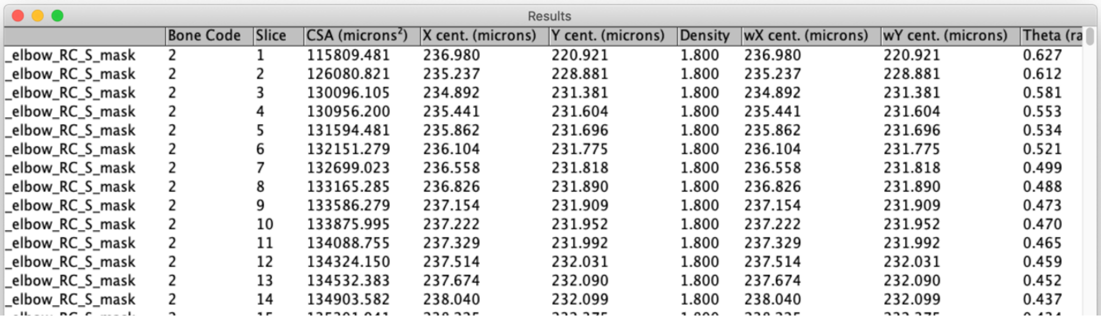

Results

	Bone Code	Slice	CSA (microns^2)	X cent. (microns)	Y cent. (microns)	Density	wX cent. (microns)	wY cent. (microns)	Theta (ra
_elbow_RC_S_mask	2	1	115809.481	236.980	220.921	1.800	236.980	220.921	0.627
_elbow_RC_S_mask	2	2	126080.821	235.237	228.881	1.800	235.237	228.881	0.612
_elbow_RC_S_mask	2	3	130096.105	234.892	231.381	1.800	234.892	231.381	0.581
_elbow_RC_S_mask	2	4	130956.200	235.441	231.604	1.800	235.441	231.604	0.553
_elbow_RC_S_mask	2	5	131594.481	235.862	231.696	1.800	235.862	231.696	0.534
_elbow_RC_S_mask	2	6	132151.279	236.104	231.775	1.800	236.104	231.775	0.521
_elbow_RC_S_mask	2	7	132699.023	236.558	231.818	1.800	236.558	231.818	0.499
_elbow_RC_S_mask	2	8	133165.285	236.826	231.890	1.800	236.826	231.890	0.488
_elbow_RC_S_mask	2	9	133586.279	237.154	231.909	1.800	237.154	231.909	0.473
_elbow_RC_S_mask	2	10	133875.995	237.222	231.952	1.800	237.222	231.952	0.470
_elbow_RC_S_mask	2	11	134088.755	237.329	231.992	1.800	237.329	231.992	0.465
_elbow_RC_S_mask	2	12	134324.150	237.514	232.031	1.800	237.514	232.031	0.459
_elbow_RC_S_mask	2	13	134532.383	237.674	232.090	1.800	237.674	232.090	0.452
_elbow_RC_S_mask	2	14	134903.582	238.040	232.099	1.800	238.040	232.099	0.437
[illegible]	[illegible]	[illegible]	[illegible]	[illegible]	[illegible]	[illegible]	[illegible]	[illegible]	[illegible]

A window with a table of results will also appear, make sure the results are in microns, not pixels. **<u>Save the table of results as a SliceGeom_mask.csv file</u>** (or another filename of your choice) for further processing and visualization in MATLAB.

Figure 3E. Cell segmentation and object counting.

This is the trickiest part of the multiscale data analysis and there is other specialized software that will likely perform much better this specific task, than the workflow outlined here. But they require time and effort to Promising candidates are the open-source CellProfiler [9], Cellpose [10] and StarDist [11]. Licensed software like Arivis also produces good results.

Our goal for this work was to keep the whole multiscale pipeline simple and minimize the amount of software used. For this reason, we aimed at using available plugins in Fiji for our cell-level analysis. Even so, there are several alternatives within Fiji. Plugins that allow for 3D analysis include BoneJ, 3D ImageJ Suite, MorphoLibJ (IJPB-plugins), 3D Objects Counter, Particle Analyzer (3D)… Check out the documentation:

https://imagej.net/BoneJ

https://imagej.net/3D_ImageJ_Suite

https://imagej.net/MorphoLibJ

https://imagej.net/3D_Objects_Counter

(Particle Analyzer (3D) currently doesn't have documentation on the ImageJ website)

Here we describe the steps we followed to segment and count the EdU-positive cells.

- Use the Canny edge detector (*Plugins › 3D › 3D Edge and Symmetry Filter*) to detect the outline of the EdU cells. Apply the filter on the masked image. Untick all options and use alpha canny equal to 0.75. The smaller its value, the smoother the edges.

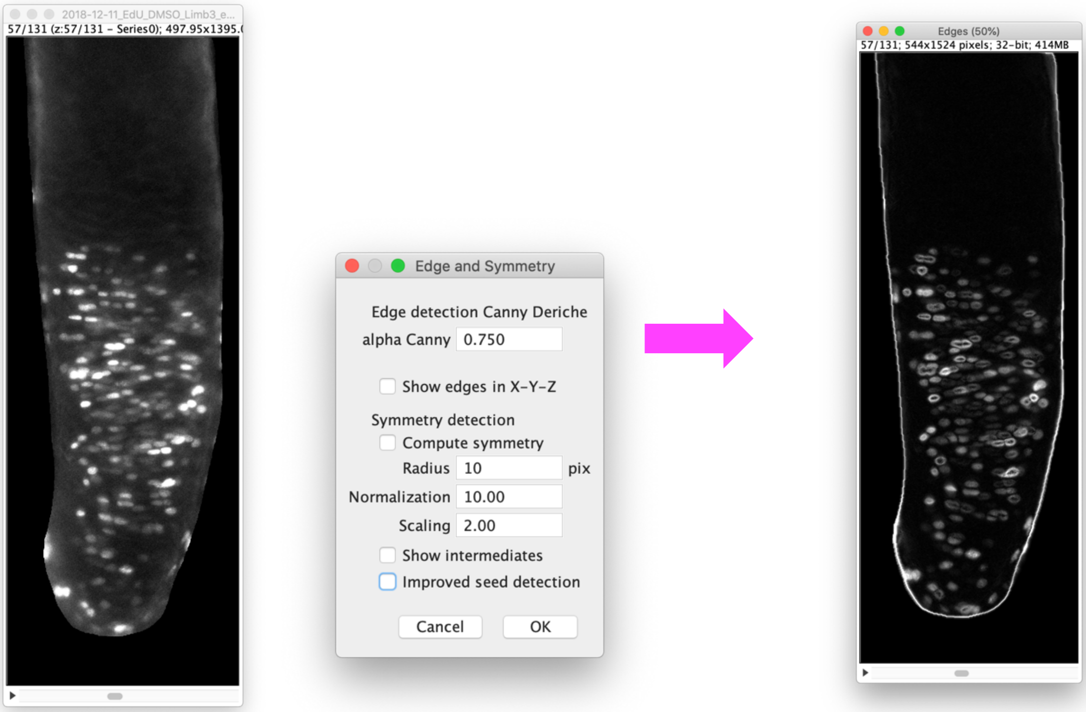

- We will subtract the "outlines" from the original image, to help better "isolate" the cells we are trying to segment. Before that, we subtract the background from the original masked image. Go to (*Process › Subtract Background...*) and use a rolling ball radius of 30 pixels. Process all the stack.

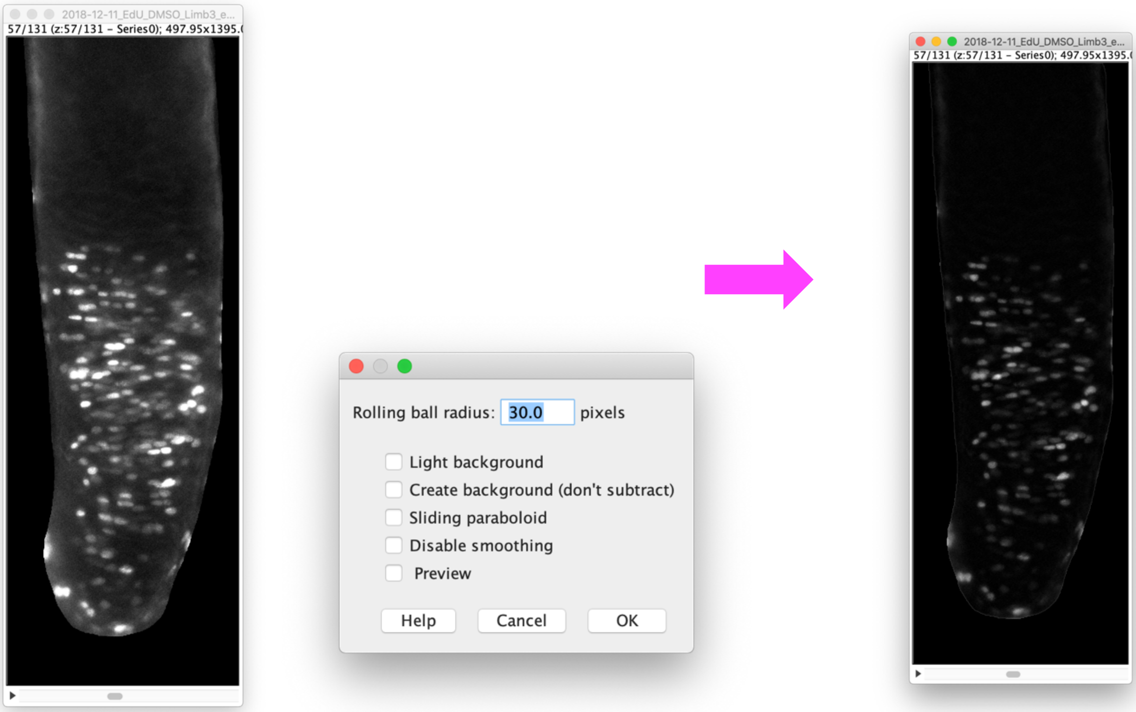

- Use the Image Calculator (*Process › Image Calculator...*) to subtract the edges from the no background image. Select the 32-bit result, because this subtraction will create negative-valued pixels and a 16-bit image cannot handle this properly.

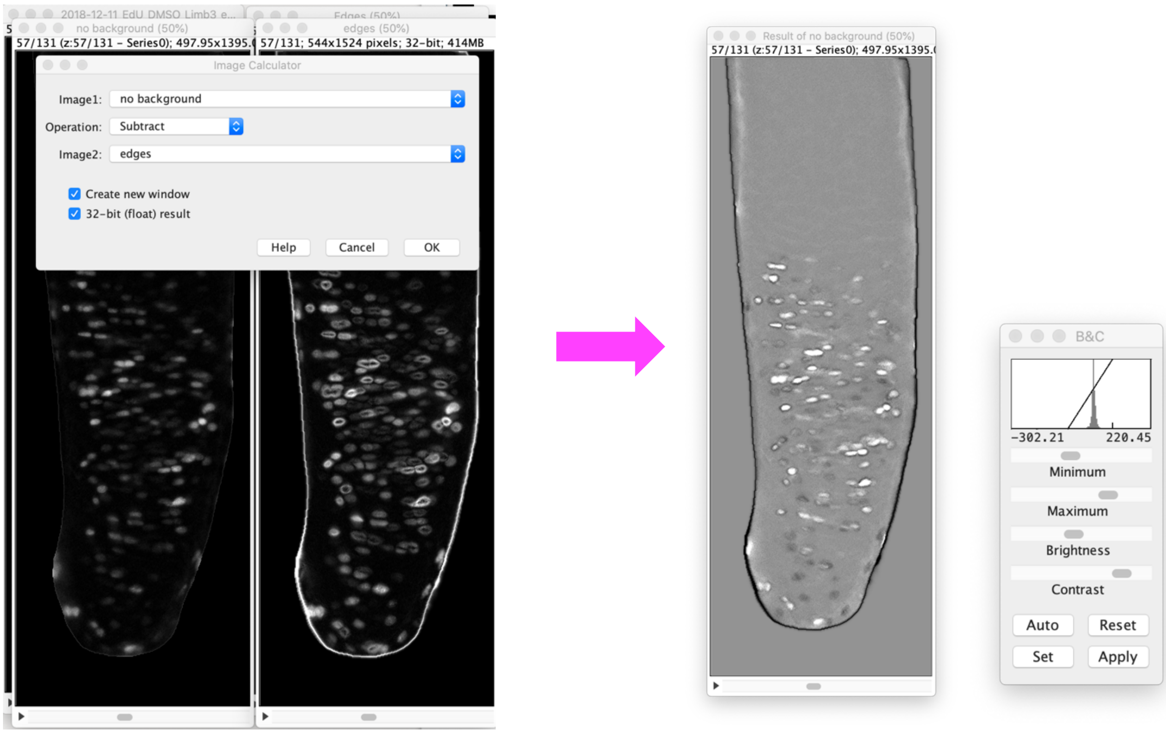

- Remove the negative-valued pixels in the B&C window (*Image › Adjust › Brightness/Contrast...*) by clicking *Set* and introducing a "0" minimum value and a "650000" max value. Then convert the image to 16-bit (*Image › Type › 16-bit*).

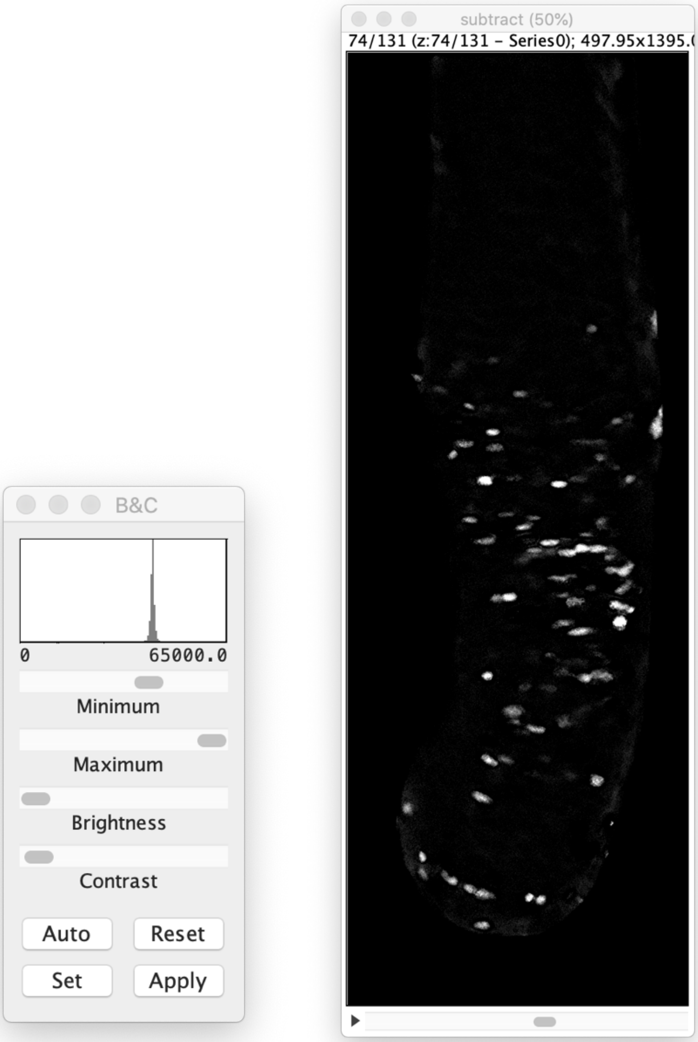

- We use this image to train the Weka Segmentation 3D plugin [12] (*Plugins › Segmentation › Trainable Weka Segmentation 3D*). Check out the documentation: https://imagej.net/Trainable_Weka_Segmentation

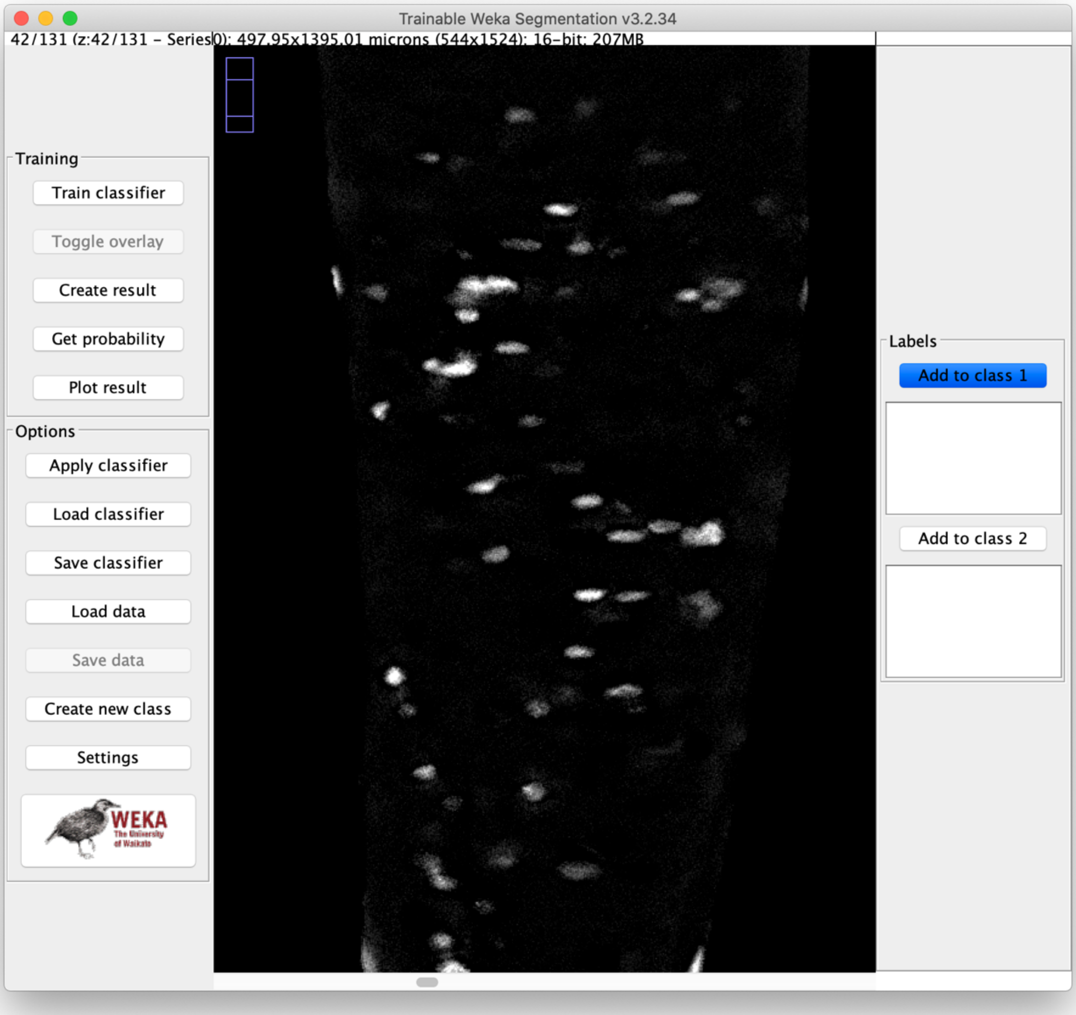

We saved the classifier we trained, so you can click on *Load classifier* and then *Create results*. The results produced should be similar to the ones we obtained:

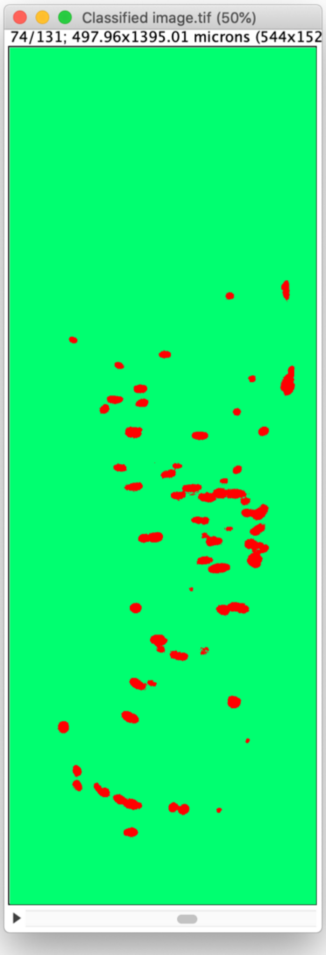

In case you want to train the classifier from scratch, or retrain the loaded classifier because the results are unsatisfactory, we give a few quick tips next. However, check out online resources for a more comprehensive description of how this plugin works.

Select the brush tool in the Fiji tool box (right click on the second box from the left shown below and select “brush tool” in the dropdown menu), and mark the center of the cells that need to be segmented. Use the “shift” key to select in multiple places of a same slice. Then click on *Add to class 1*. Class 1 will be the cells we want to segment. Scroll through the stack and mark different cells throughout. Use the “hand” (circled below) to move up and down in each slice.

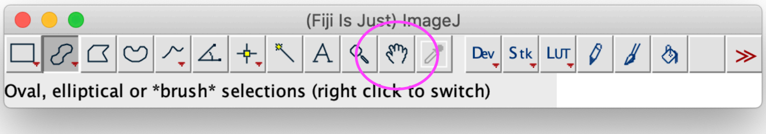

Then, repeat but with the background. Add this selection to *Add to class 2*. Use other tools like line or bean tool if needed.

Tip: It’s important to mark a variety of cell types and background types, rather than a huge quantity. In fact, if you add too many pixels to the classifier, it will crash and the training will not work. It’s better to train it first with few but comprehensive pixels added to the classes. For example, mark 3 or 4 cells every 10-20 stacks, trying to capture a variety of cell positions, shapes and intensities. Mark a bit of background outside the actual humerus segmentation and within the humerus, so the algorithm knows to recognize the uneven light staining within the humerus as background. Also mark with a line or brush cell divisions. Start with an initial training like this, and then keep retraining until we get the results we desire. Here is an example of different strokes we can use for the training:

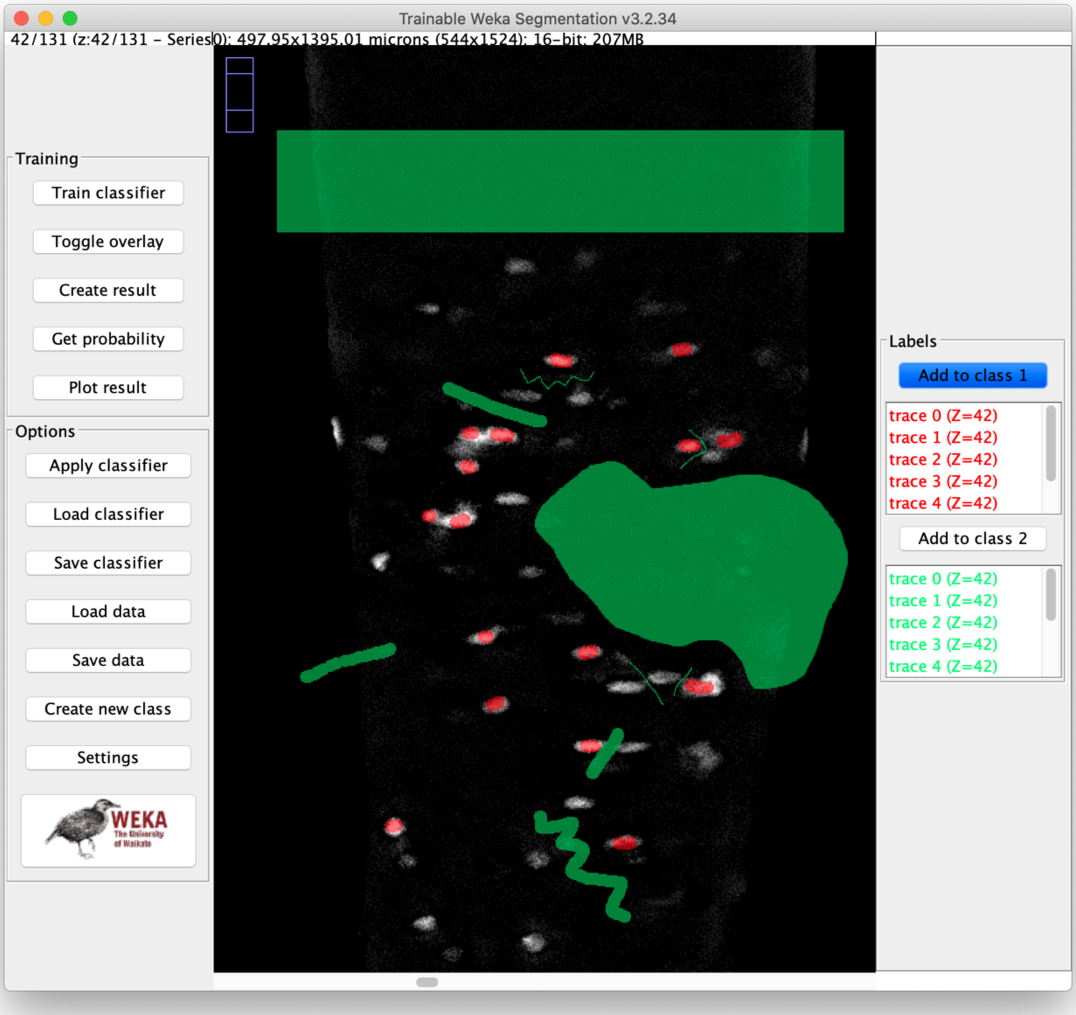

When done with the selection, click on *Train classifier* and wait (patience!). Then, click on *Create result* and wait again. Repeat with *Get probability*. Save the classifier.

- Convert the green and red results stack to an 8-bit grayscale (*Image › Type › 8-bit*) and threshold the image (*Image › Adjust › Threshold...*). We need to do this to be able to apply the Fill holes filter.

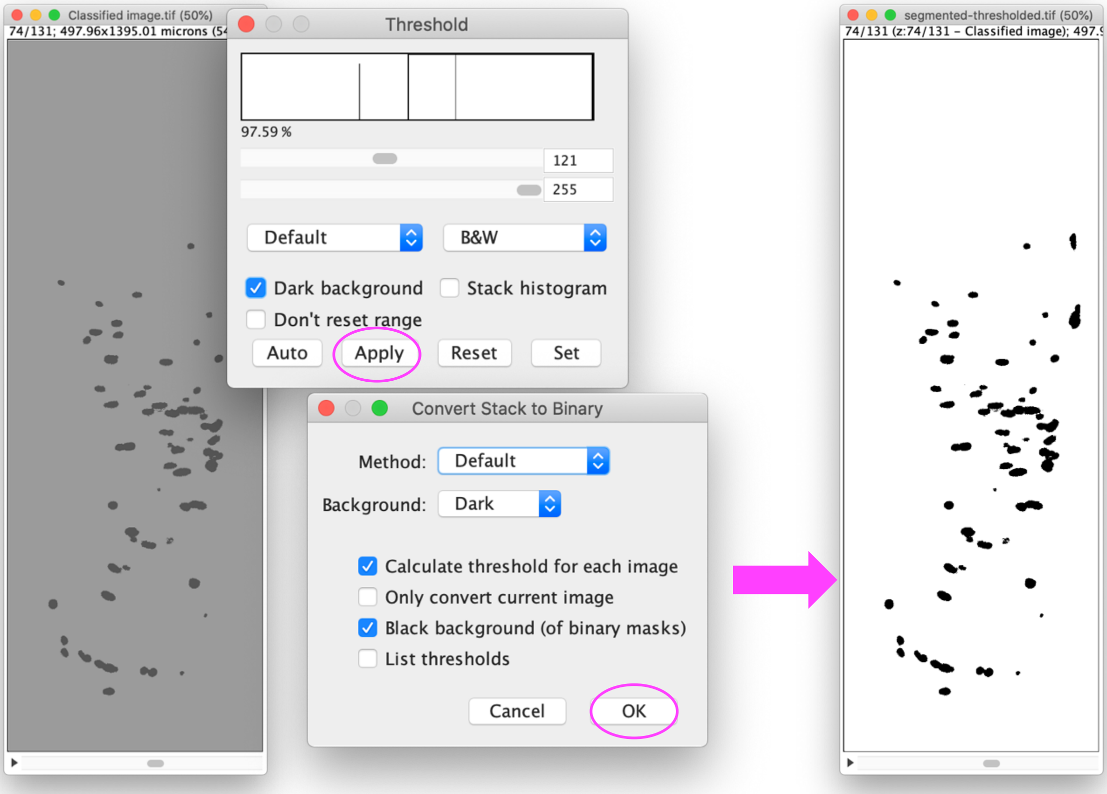

- Fill holes using the 3D ImageJ Suite filter (*Plugins › 3D › 3D Fill Holes*). Before that, we need to invert the binarized image so that the cells are white (255 intensity value) and the background is black (0 intensity value). For that, apply the invert command (*Edit › Invert*).

  Note: For our image, the cells were mostly "homogeneously filled" already, so maybe we could have skipped this filter. Only a handful of cells had some "empty" space inside, for example:

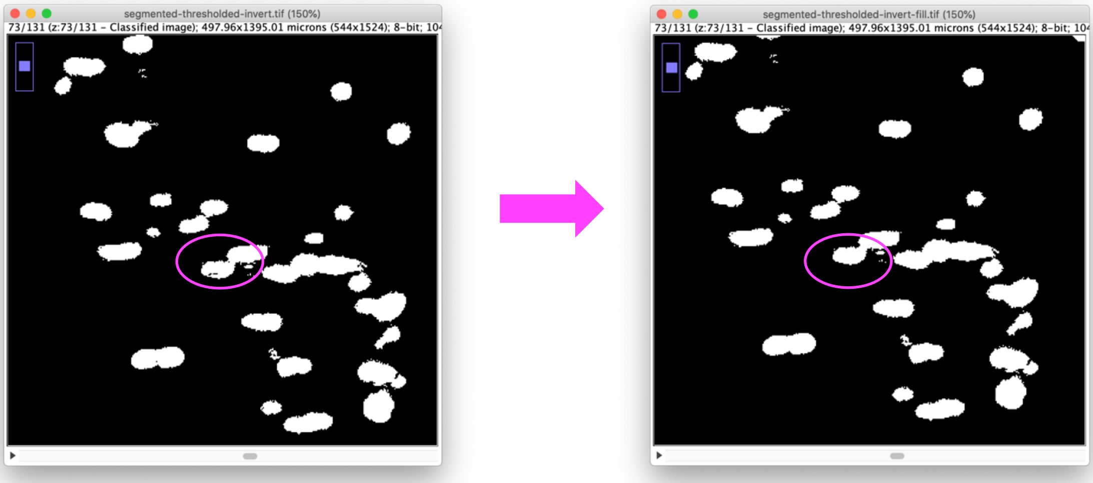

- Blur and split cells. We want to smooth the rugged edges of cells and remove “floating pixels” by blurring. Then, we want to split the “blobs” of cells stuck together into separate objects. The aim of these two filters is to improve the results when we count the segmented objects, i.e. small clusters of pixels should not be counted as a cell, and large blobs should not be a single cell, but rather separate ones.

  For this, we apply first the Gaussian Blur 3D (*Process › Filters › Gaussian Blur 3D...*). A larger sigma value will blur the cells more: this removes the “floating pixels” better, but also makes it harder to split the blobs into separate cells because their irregular shape is smoothed out. We select a value of 0.8 (although 0.6 also seems to work well).

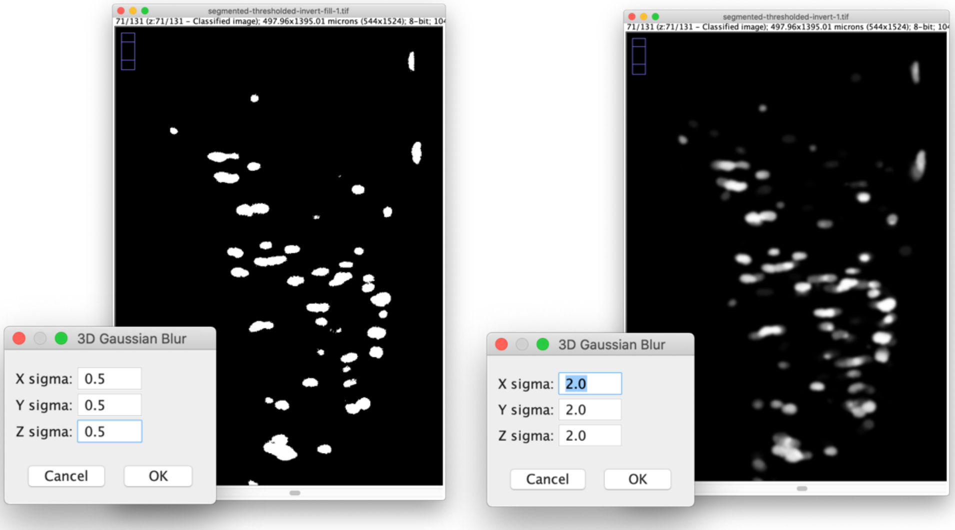

  Then, we apply a 3D Watershed Split (*Plugins › 3D › 3D Watershed Split*) to separate the blobs into distinct cells. Here, we use automatic seeds and must select the appropriate radius. A value of 10 pixels worked well (8 pixels produced also good results).

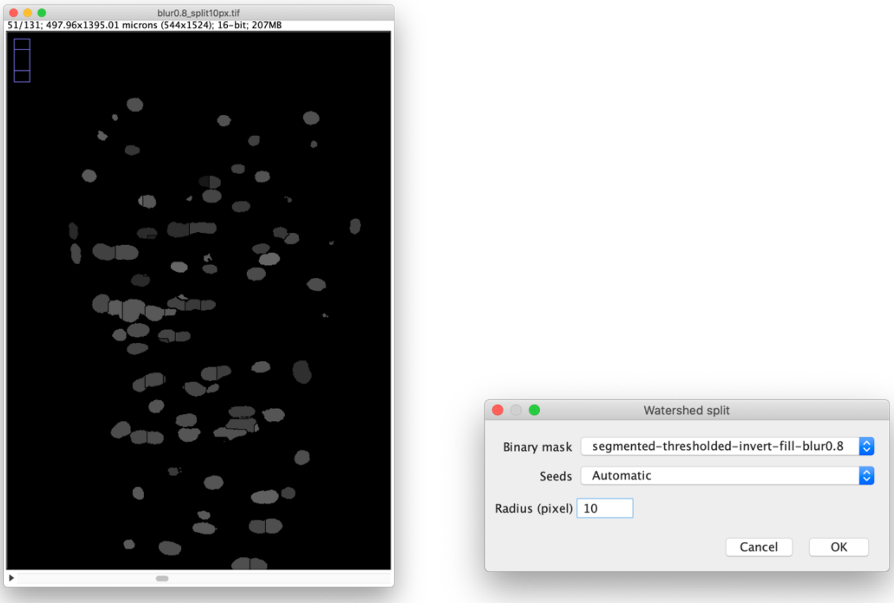

Note: there is much room for improvement in these results. Dedicating time to try out different combinations of parameters for the Gaussian Blur and Watershed Split, and also going back to retrain the Weka Segmentation algorithm would help improve these results. For our demonstrative purposes, these results seem reasonable and we have moved on to the next step.

Tip: double check the properties! It seems that applying the 3D Watershed Split results in loss of pixel resolution. Introduce the correct unit of length and pixel width, height and depth, we need them for the Object Counter properties to be correct.

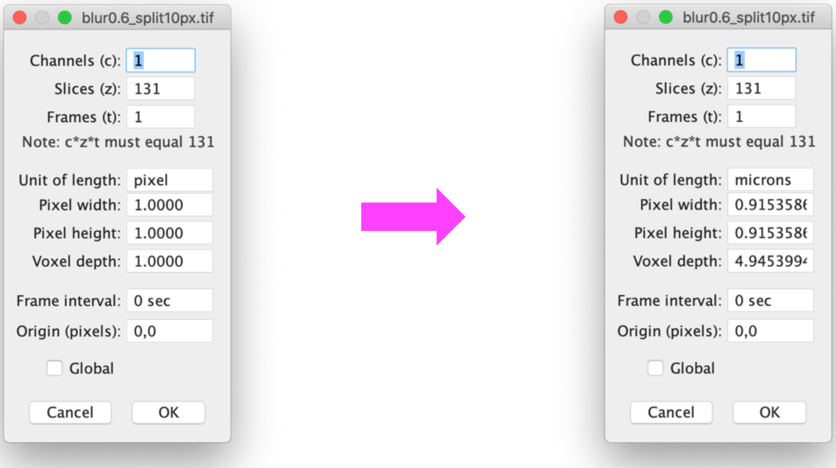

- Run the 3D Objects Counter [13] (*Analyze › 3D Objects Counter*). We set a threshold of 1. This is because the Watershed Split simply colored each object with a constant intensity value. So, any value different than black (0 intensity) will belong to an object in our case. We also set a minimum size of our objects to 1000. This is a rough estimate (a cube of sides with 10 pixels of length) to ensure that, in the unlikely case there are still "floating pixels" not eliminated by the Gaussian Blur, we don't count them. Scroll through the slices using the "Slice" slider, to make sure all cells are marked in red before clicking *Run*.

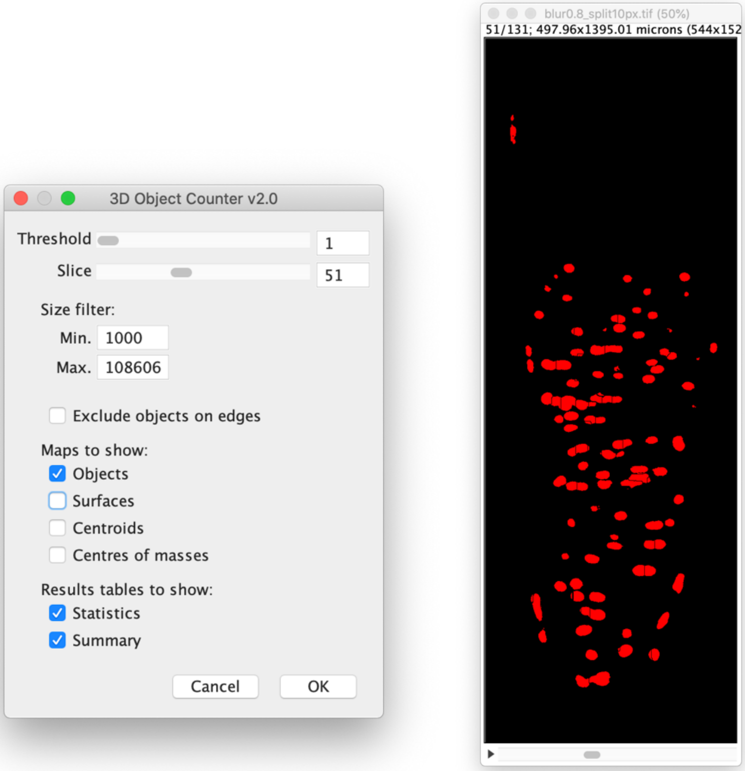

A window with a table of results will appear, make sure the results are in microns, not pixels. **<u>Save the table of results as a 3DObjectCount.csv file</u>** (or another filename of your choice) for further processing and visualization in MATLAB.

- To obtain the surface of the segmented cells, we follow exactly the same steps as we did for the surface of the humerus. Using the results from the Watershed Split filter (ensure the properties have been updated!), open the 3D Viewer plugin. Then export the surface as an .stl. First, convert the volume to a surface (*Edit › Display As… › Surface*) and then export (*File › Export Surfaces … › STL (binary)*). We can use MeshLab to smooth out the cell surfaces and reduce the mesh (and file) size, if needed for visualization purposes.

  <u>Save the cell-surfaces.stl file</u> (or another filename of your choice) for visualization in MATLAB.

Figure 3F. Reslice and mask the rotated cropped image, and analyze the pixel maps.

- Import the EdU channel stack that is rotated, cropped and substacked, but NOT masked. To visualize the EdU intensity in 3D, create a duplicate stack and then adjust (and apply) the threshold values in Brightness and Contrast. Then open 3D Viewer. Background color, bounding box, threshold, etc. can be adjusted in the plugin. Threshold adjustment in the 3D Viewer doesn't give exactly the same results as adjusting (and applying the changes) in the stack before opening.

Note: We need to mask again the image because now we will have to set the background to the maximum intensity value possible, not to zero. This is because we could potentially have "black" (0 intensity) pixels inside our humerus, and then our code would not be able to distinguish the ones inside the humerus from the ones outside. We will (should) never have maximum intensity pixels in our stack if image acquisition was done correctly. So, our code will know to disregard those pixels, which we artificially introduced, and we can be sure that they always correspond to background.

- Import the mask that is NOT resliced.

- Run the "ImageJ_reslice_and_intensity_count_16bits.ijm" script. To do so, drag and drop the file on the Fiji tool bar. A window will open with the code. Click on *Run* and follow the instructions. When a window pops up requesting that you load an image, BEFORE clicking "OK" either select (activate) the window with the image required or load the image. Then, click *OK*.

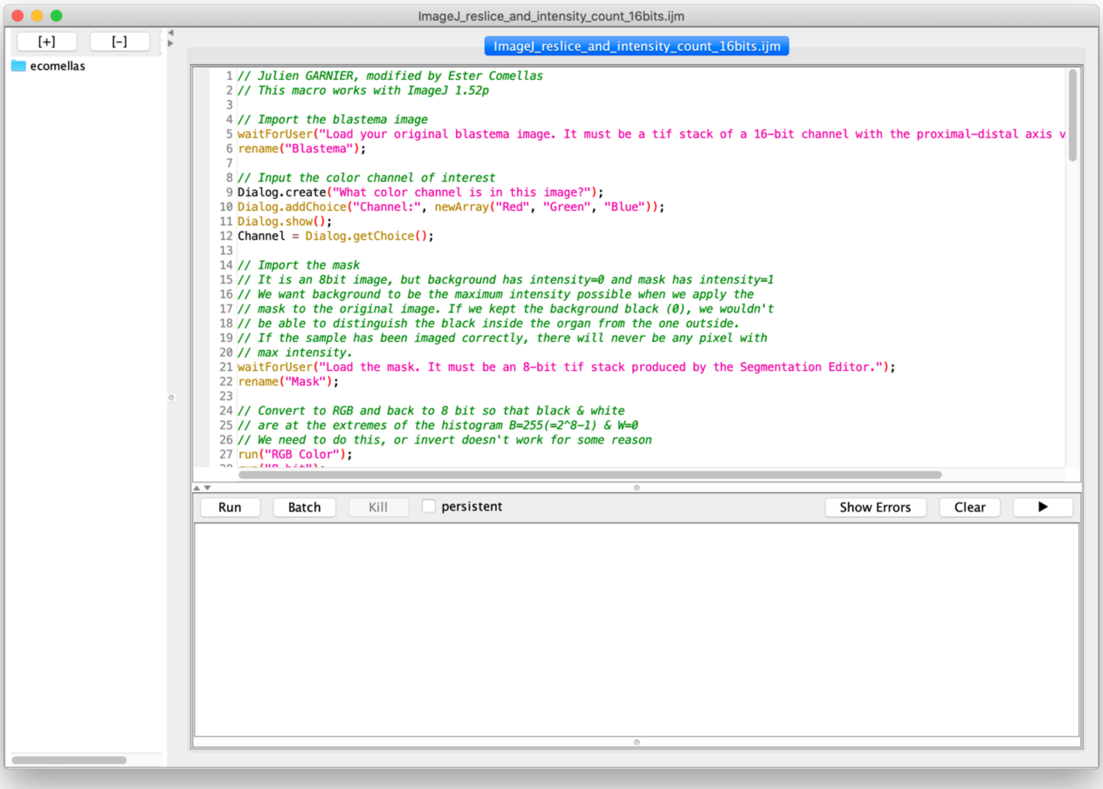

Note: This script (i) masks the rotated and cropped image, as explained above; (ii) reslices along the vertical axis (which should correspond to the proximodistal axis); and (iii) prints a table with the pixel intensity data and statistics of each cross-sectional slice (including, the background, which will be max intensity).

The code prompts you to **save the table of results as a ImageJ-results-intensity.csv file** (or another filename of your choice) for further processing and visualization in MATLAB.

Figure 4 results

The MATLAB code "MultiscaleDataAnalysis.m" uses as input all the files generated in the previous steps and produces a series of plots to quantify the bone rudiment at organ, cellular and molecular levels. Check out the comments in the code for a detailed description of how the data obtained with Fiji is processed and visualized. Make sure to update the "user input" section in the code with your filenames and other image-dependent parameters.

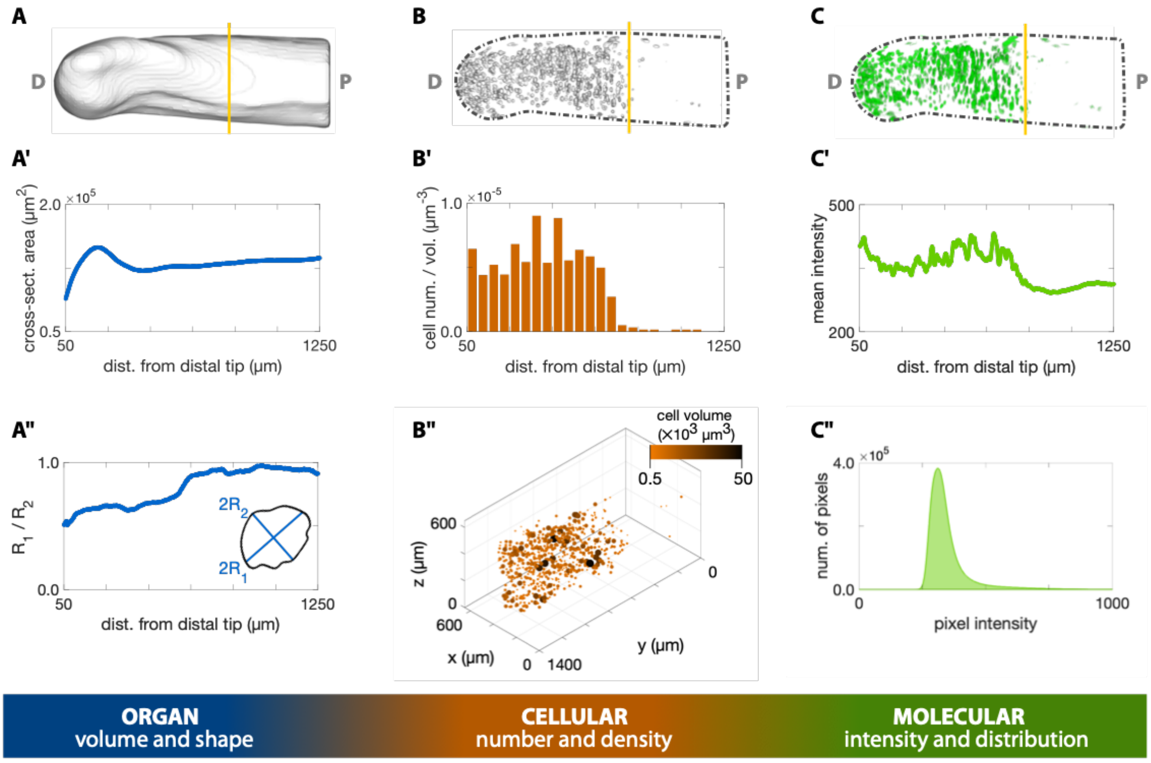

References

[1] J. Schindelin *et al.*, 'Fiji: An open-source platform for biological-image analysis', *Nat. Methods*, vol. 9, no. 7, pp. 676–682, Jul. 2012.

[2] MATLAB, 'version 9.7.0.813654 (R2019b)'. The MathWorks Inc., Natick, Massachusetts, 2019.

[3] M. Linkert *et al.*, 'Metadata matters: Access to image data in the real world', *J. Cell Biol.*, vol. 189, no. 5, pp. 777–782, 2010.

[4] I. G. Goldberg *et al.*, 'The Open Microscopy Environment (OME) Data Model and XML file: open tools for informatics and quantitative analysis in biological imaging.', *Genome Biol.*, vol. 6, no. 5, p. R47, May 2005.

[5] C. A. Schneider, W. S. Rasband, and K. W. Eliceiri, 'NIH Image to ImageJ: 25 years of image analysis', *Nat. Methods*, vol. 9, no. 7, pp. 671–675, Jul. 2012.

[6] B. Schmid, J. Schindelin, A. Cardona, M. Longair, and M. Heisenberg, 'A high-level 3D visualization API for Java and ImageJ', *BMC Bioinformatics*, vol. 11, pp. 274–280, 2010.

[7] P. Cignoni, M. Callieri, M. Corsini, M. Dellepiane, F. Ganovelli, and G. Ranzuglia, 'MeshLab: An open-source mesh processing tool', in *6th Eurographics Italian Chapter Conference 2008 - Proceedings*, 2008, pp. 129–136.

[8] M. Doube *et al.*, 'BoneJ: Free and extensible bone image analysis in ImageJ', *Bone*, vol. 47, no. 6, pp. 1076–1079, 2010.

[9] C. McQuin *et al.*, 'CellProfiler 3.0: Next-generation image processing for biology', *PLOS Biol.*, vol. 16, no. 7, p. e2005970, Jul. 2018.

[10] C. Stringer, T. Wang, M. Michaelos, and M. Pachitariu, 'Cellpose: a generalist algorithm for cellular segmentation', *bioRxiv*, p. 2020.02.02.931238, Jan. 2020.

[11] U. Schmidt, M. Weigert, C. Broaddus, and G. Myers, 'Cell detection with star-convex polygons', in *Lecture Notes in Computer Science (including subseries Lecture Notes in Artificial Intelligence and Lecture Notes in Bioinformatics)*, 2018, vol. 11071 LNCS, pp. 265–273.

[12] I. Arganda-Carreras *et al.*, 'Trainable Weka Segmentation: A machine learning tool for microscopy pixel classification', *Bioinformatics*, vol. 33, no. 15, pp. 2424–2426, 2017.

[13] S. Bolte and F. P. Cordelières, 'A guided tour into subcellular colocalization analysis in light microscopy', *Journal of Microscopy*, vol. 224, no. 3. John Wiley & Sons, Ltd, pp. 213–232, 01-Dec-2006.
