# Supplementary figures and images for: 3D visualization of macromolecule synthesis
Source: eLife. 2020 Oct 14;9:e60354. doi: 10.7554/eLife.60354 (PMC7669265; doi:10.7554/eLife.60354)

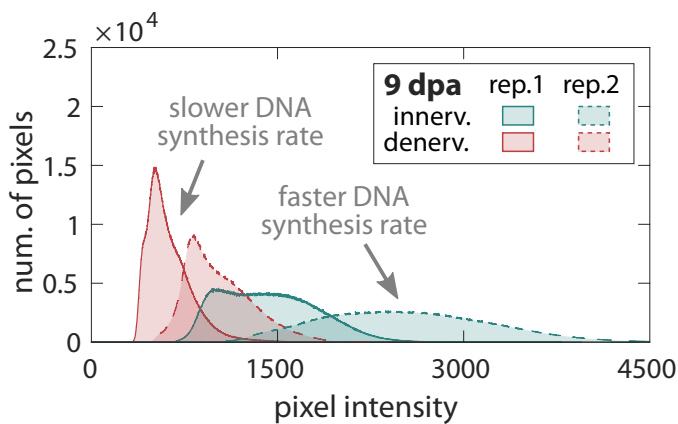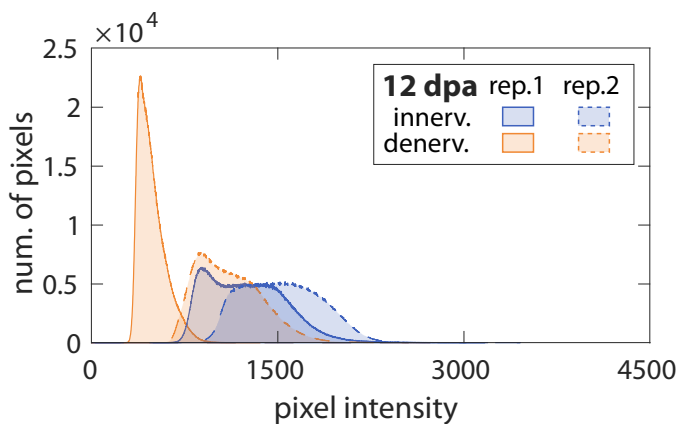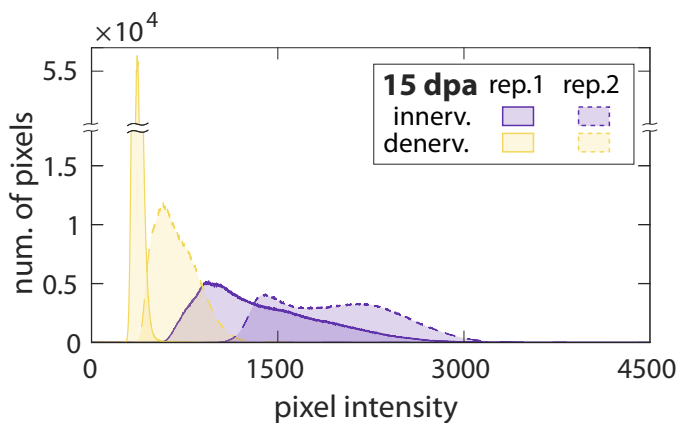

Supplement: Source code 2. — Source code used in the 3D quantification of DNA synthesis in innervated/denervated regenerating limbs depicted in Figure 6. Annotated ImageJ and Matlab scripts are provided. The raw images are available at Northeastern University’s Digital Repository and also upon request to the authors. [file elife-60354-code2.zip › Source code file 2/Fig6_suppl_v1.pdf]

**A**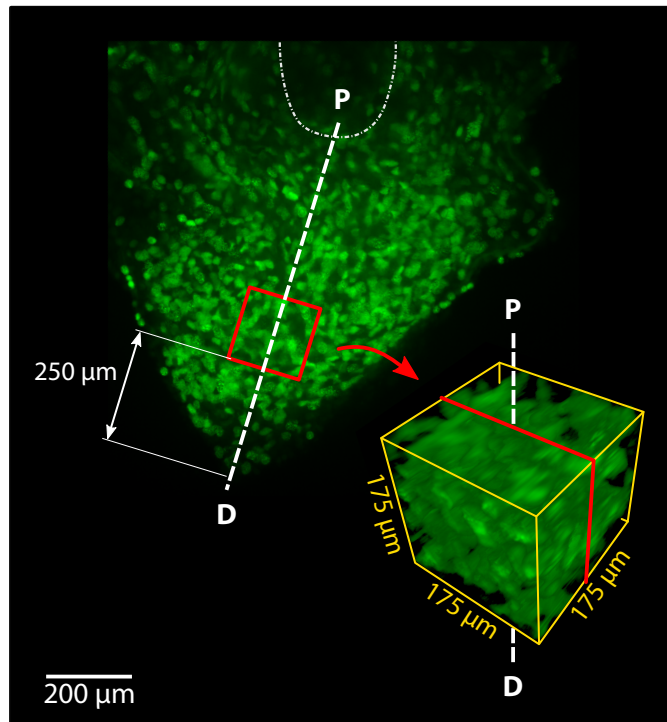**B**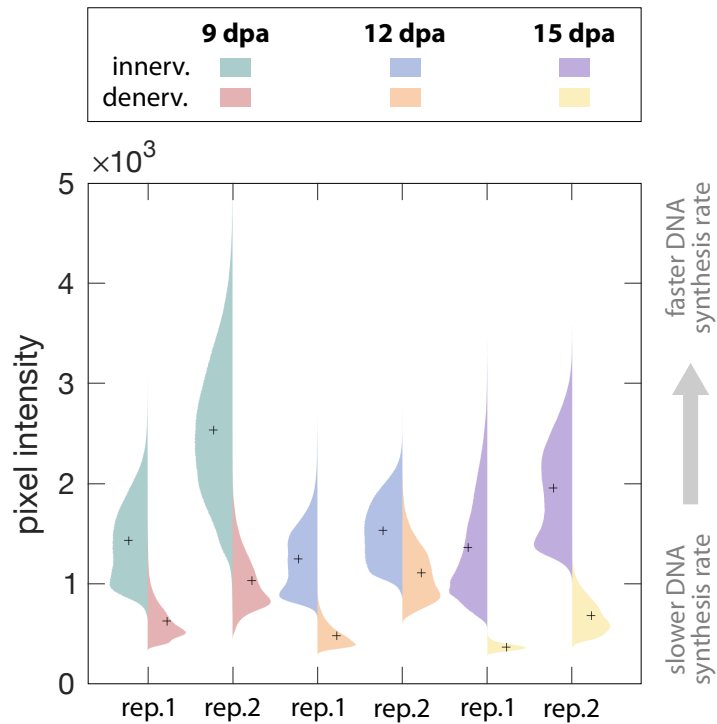

Supplement: Source code 2. — Source code used in the 3D quantification of DNA synthesis in innervated/denervated regenerating limbs depicted in Figure 6. Annotated ImageJ and Matlab scripts are provided. The raw images are available at Northeastern University’s Digital Repository and also upon request to the authors. [file elife-60354-code2.zip › Source code file 2/Fig6_v7.pdf]
